# Management of upper gastrointestinal bleeding from portal hypertension: Elective or emergency operation?

**DOI:** 10.12669/pjms.303.4520

**Published:** 2014

**Authors:** Sen Liu

**Affiliations:** 1 Sen Liu, MD.Department of General surgery,Tianjin 4th Centre Hospital,Tianjin, 300140, China.

**Keywords:** Upper gastrointestinal bleeding, Portal hypertension, Splenectomy, Periesophagogastric devascularization, Elective operation, Emergency operation

## Abstract

***Objective***: To evaluate the clinical outcome of emergency and elective operation of splenectomy with periesophagogastric devascularization in treating upper gastrointestinal hemorrhage resulted from portal hypertension.

***Methods***: We retrospectively reviewed 219 patients of upper gastrointestinal hemorrhage resulted from portal hypertension treated using emergency or elective operation between Jul 2002 and Aug 2010. The clinical data were collected and analyzed.

***Results***: In the group of elective operation, four patients with grade B and three with grade C died, and in the group of emergency operation, two patients with Grade B and four with Grade C died. The Grade C patients treated using emergency operation presented with a higher mortality than those treated using elective operation, but no significant difference was found (p>0.05). In the two groups, no patients with Grade A died. 17 cases (11.1%) suffered from complications in the group of elective operation and 11 cases (16.7 %) in emergency operation (p>0.05). The complication rate in patients with Grade C is significantly higher than that in patients with Grade A or B in each group (p<0.05). The hospital stay and cost in group of elective operation are significantly higher than those in group of emergency operation (p<0.05).

***Conclusion***: The patients with Grade A or B treated using emergency operation have similar clinical outcomes as those treated using elective operation, but emergency operation may result in higher rate of death and complication in patients with Grade C.

## INTRODUCTION

Upper gastrointestinal hemorrhage is one of the life-threatening complications resulted from portal hypertension. Development of gastroesophageal varices and variceal hemorrhage are the most direct consequence of portal hypertension.^1^ When variceal bleeding is suspected, patients should be hemodynamically stabilized, vasopressors, antibiotic^2^^,^^3^ and endoscopic therapy^4^ can be performed to control the hemorrhage. However, when medical or endoscopic control of bleeding was not achievable, surgical procedures are needed.^5^

 Splenectomy with periesophagogastric devascularization is often performed for the patients with upper gastrointestinal bleeding resulted from portal hypertension. In terms of operation opportunity, many scholars suggested the elective operation should be performed because the emergency operation can increase the mortality and incidence of complications.^6^ The hemorrhagic shock and organs hypoperfusion may result in the mortality during emergency operation as high as 30%.^7^ Conversely, some scholars advocated the upper gastrointestinal hemorrhage should be treated using emergency operation, because the acute bleeding may aggravate shock, damage liver and renal function, and lead to a more higher mortality^8^ if the patients can’t avoid the surgical management at last. To the best of our knowledge, up to now, no agreement is reached on the selection of operation opportunity for the treatment of upper gastrointestinal bleeding resulted from portal hypertension.

 Therefore, in the current study, we retrospectively reviewed 219 patients of upper gastrointestinal hemorrhage resulted from portal hypertension treated using emergency or elective operation between Jul 2002 and Aug 2010. The aim of the current study was 1) To compare the clinical outcomes of emergency operation and elective operation; 2) To help surgeons make strategies for the treatment of upper gastrointestinal bleeding resulted from portal hypertension.

## METHODS

***General data: ***Two hundred and nineteen patients of upper gastrointestinal hemorrhage resulted from portal hypertension treated in our hospital from July 2002 to August 2010 were reviewed retrospectively. The data including patient’s age, medical history, hospital stay, hospital cost, postoperative complications, wound infection, postoperative rebleeding, subphrenic infection and mortality rate were collected. The current study was approved by the ethics committee of our hospital.

Our study population consisted of patients diagnosed with upper gastrointestinal hemorrhage resulted from portal hypertension and treated in emergency or elective operation, and the cases of upper gastrointestinal hemorrhage resulted from other reasons were excluded. In 219 cases, 139 cases were male and 80 female, with a mean age of 48.9 years old (ranged from 31 to 81 years old). One hundred and fifty-three cases were included in the group of elective operation and 66 in the group of emergency operation. According to Child classification, 88 cases were Grade A, 45 were grade B and 20 were Grade C in the group of elective operation; 36 cases were grade A, 19 were grade B and 11 were Grade C in the group of emergency operation. There is no significant difference in medical history, age, gender and classification between the two groups.

***Operative methods: ***The following surgery was performed as described in detail by Zhang.^9^ Under general anesthesia, a left subcostal incision was used to perform the routine splenectomy, then the right gastric vein was disconnected near the gastric angular incisura, followed by the disconnection of the gastric branch of the right gastric vein as well as the branches of the gastric coronary veins. The esophageal branch was disconnected and suture-ligated up to 7-9 cm of the esophageal inferior segment, followed by the disconnection of the high esophageal branch. The gastric posterior veins, short gastric veins and the left subphrenic vein were ligated with sutures. In addition, the left gastric artery, left gastroepiploic artery, gastric posterior artery and left subphrenic artery were also disconnected.

***Statistical Analysis: ***Statistical analysis was performed using SPSS17.0 (SPSS Inc., Chicago, IL, USA). Depending on the characteristics of the variables being compared, independent 2-sample t test was applied to compare the difference of measurement data between two groups, a chi-square test were used to compare the difference of enumeration data between two groups. A probability value of < 0.05 was considered to indicate statistical significance.

## RESULTS

***Postoperative complications: ***The details of the complications in two groups are listed in Table-I. 17 cases (11.1%) suffered from complications in group of elective operation and 11 cases (16.7 %) in group of emergency operation (p>0.05). In the group of elective operation, the complication rate in patients with Grade A, B and C is 6.8%, 8.9% and 35%, in group of emergency operation is 5.6%, 10.5% and 54.5%, respectively. No significant difference in complication rate was found in patients with the same classification between the two groups (p>0.05), but the complication rate in patients with Grade C is significantly higher than that in patients with Grade A or B in each group (p<0.05).

***Postoperative mortality: ***The mortality of two groups is listed in Table-II. No patients with Grade A died. In the group of elective operation, four patients with grade B and three patients with grade C died. In the group of emergency operation, two patients with Grade B and four patients with Grade C died. The cause of death is blood loss and DIC in three cases, multiple system organ failure in four cases and hepatic failure in six cases. The Grade B patients have the similar mortality in two groups (p>0.05). The group C patients have a higher mortality in the group of emergency operation than elective operation, but no significant difference was found (p>0.05). In addition, the mortality in Grade C patients is higher than that in Grade B patients in both groups, while no significant difference was found(p>0.05).

***Hospital stay and cost: ***The mean hospital stay in the group of emergency operation is 11.8±4.9 days and hospital cost is 16787.80± 5491.67 yuan; while in the group of elective operation, the mean hospital stay is 19.7±5.3 days and hospital cost is 23019.87±6982.03 yuan. The mean hospital stay and cost in the group of elective operation are significantly higher than those in the group of emergency operation (p<0.05).

## DISCUSSION

In the current study, we performed a retrospective review of our experience in the treatment of upper gastrointestinal hemorrhage resulted from portal hypertension, to help general surgeons better determine the treatment strategy of the fatal condition.

**Table-I:** The complications after surgery in two groups

*Complications*	*Elective operation*				*Emergency operation*		
	*Grade A*	*Grade B*	*Grade C*		*Grade A*	*Grade B*	*Grade C*
Subphrenic infection	1	1	1		0	0	2
Pleural effusion	0	0	0		1	0	0
Wound infection	2	1	0		0	1	0
Intraperitoneal hemorrhage	1	0	2		0	0	1
Ascites	0	0	1		1	0	1
Portal thrombosis	2	1	0		0	0	0
Hepatic failure	0	1	3		0	1	2

**Table-II:** The mortality of elective operation and emergency operation

	*Grade A*	*Grade B*	*Grade C*
Elective operation	0	8.9%	15%
	0/88	4/45	3/20
Emergency operation	0	10.5%	36.4%
	0/36	2/19	4/11

In terms of the treatment of upper gastrointestinal hemorrhage resulted from portal hypertension, the initial approach is a combination of vasoactive drugs, antibiotics and endoscopic therapy.^10^ However, sometimes these methods can not resolved the problems completely. The use of balloon tamponade, as only a bridging procedure, has been performed by some surgeons, while its serious drawback such as necrosis, rupture of the esophagus and aspiration pneumoniathe, should only be applied by an experienced physician under fluoroscopic control.^5^ The current study demonstrated that the patients with Grade A or B treated using emergency operation presented the similar mortality and complication rate as those treated using elective operation. As a result, we suggest that when the bleeding can’t be controlled, emergency operation should be performed as soon as possible for patients with Grade A or B, which is important to save the lives. In addition, we found, in the current study, the mean hospital stay and cost in the group of elective operation are significantly higher than those in the group of emergency operation, which is also the advantage of emergency operation.

Moreover, in the current study, we found the patients with Grade C presented with 35% and 54.5% of complication rate, and 15% and 36.4% of mortality in the group of elective and emergency operation respectively. The death and complication rate in patients with Grade C are higher than those in Grade A or B, demonstrating that the survival of patients who have undergone surgery is dependent on liver function.^5^ In the viewpoints of Tiuca and Sztogrin, the patients with esophageal varices usually have severe liver disease and thus suffer from poor nutrition, blood clotting disorders or encephalopathy, all of which can adversely affect morbidity and mortality.^11^ Bari and colleagues advocated the efficacy of surgery may vary depending on the severity of liver disease and the risk of surgical failing is very high in patients with Grade C.^1^ In addition, we found in the current study, the death and complication rate in patients with Grade C is higher in the group of emergency operation than those in the group of elective operation, which confirmed the viewpoints of the above authors. Although the difference has no significance, which may be contributed to the small sample size of grade C patients, we can conclude from the current study that the emergency operation may result in a higher risk for the patients with Grade C.

Some authors have suggested previously that the patients with poor liver function should not be operated because of high operative mortality^12^, as operation may damage the liver function further. However, in our opinion, with the development of perioperative treatment and emergency operation, we advocate that emergency operation for Grade C patients should also be performed immediately if acute gastroesophageal variceal bleeding can’t be controlled by conservative treatment, and if the patients have no contraindications.

Consequently, we concluded from the current study that the patients with Grade A or B treated using emergency operation have similar clinical outcomes as those treated using elective operation, but emergency operation may result in higher rate of death and complication in patients with Grade C, and surgeons should select proper operation opportunity according to the specific conditions and operation indications of patients. Our study has its limitations. The sample size was relatively small, especially in patients with Grade C. We believe our conclusion may be more convictive if a large scale, clinical control trial is performed on the issue. Subsequently, more study need to be carried out in the future.

## References

[1] Bari K, Garcia-Tsao G (2012). Treatment of portal hypertension. World J Gastroenterol.

[2] Fernandez J, Ruizdel Arbol L, Gomez C, Durandez R, Serradilla R, Guarner C (2006). Norfloxacin vs ceftriaxone in the prophylaxis of infections in patients with advanced cirrhosis and hemorrhage. Gastroenterology.

[3] Hou MC, Lin HC, Liu TT, Kuo BI, Lee FY, Chang FY (2004). Antibiotic prophylaxis after endoscopic therapy prevents rebleeding in acute variceal hemorrhage: a randomized trial. Hepatology.

[4] Lo GH, Lai KH, Ng WW, Tam TN, Lee SD, Tsai YT (1992). Injection sclerotherapy preceded by esophageal tamponade versus immediate sclerotherapy in arresting active variceal bleeding: a prospective randomized trial. Gastrointest Endosc.

[5] Biecker E (2013). Portal hypertension and gastrointestinal bleeding: Diagnosis, prevention and management. World J Gastroenterol.

[6] Cao W, Chen Y, Guan H Analysis of postoperative complications in patients with portal hypertension and its management. Chin J Gen Surg.

[7] Wang S The comparative observation of effect of octreotide acetate and the traditional medicine in decreasing the portal pressure. Chongqing Med.

[8] Wu J, Qiu F, Wu M (2008). Huang-jiasi Surgery.

[9] Zhang Y, Wen TF, Yan LN, Yang HJ, Deng XF, Li C (2012). Preoperative predictors of portal vein thrombosis after splenectomy with periesophagogastric devascularization. World J Gastroenterol.

[10] Banares R, Albillos A, Rincon D, Alonso S, Gonzalez M, Ruiz-del-Arbol L (2002). Endoscopic treatment versus endoscopic plus pharmacologic treatment for acute variceal bleeding: a meta-analysis. Hepatology.

[11] Tiuca N, Sztogrin W (2011). The news of treatment of variceal upper gastrointestinal bleeding. J Med Life.

[12] Xu XB, Cai JX, Leng XS, Dong JH, Zhu JY, He ZP (2005). Clinical analysis of surgical treatment of portal hypertension. World J Gastroenterol.

